# State-Dependent Decoding Algorithms Improve the Performance of a Bidirectional BMI in Anesthetized Rats

**DOI:** 10.3389/fnins.2017.00269

**Published:** 2017-05-31

**Authors:** Vito De Feo, Fabio Boi, Houman Safaai, Arno Onken, Stefano Panzeri, Alessandro Vato

**Affiliations:** ^1^Neural Computation Laboratory, Istituto Italiano di TecnologiaRovereto, Italy; ^2^Nets3 Laboratory, Department of Neuroscience and Brain Technologies, Istituto Italiano di TecnologiaGenova, Italy; ^3^Department of Neurobiology, Harvard Medical SchoolBoston, MA, United States

**Keywords:** state dependence, brain-machine interfaces, neural coding, neural response variability, information coding, network state

## Abstract

Brain-machine interfaces (BMIs) promise to improve the quality of life of patients suffering from sensory and motor disabilities by creating a direct communication channel between the brain and the external world. Yet, their performance is currently limited by the relatively small amount of information that can be decoded from neural activity recorded form the brain. We have recently proposed that such decoding performance may be improved when using state-dependent decoding algorithms that predict and discount the large component of the trial-to-trial variability of neural activity which is due to the dependence of neural responses on the network's current internal state. Here we tested this idea by using a bidirectional BMI to investigate the gain in performance arising from using a state-dependent decoding algorithm. This BMI, implemented in anesthetized rats, controlled the movement of a dynamical system using neural activity decoded from motor cortex and fed back to the brain the dynamical system's position by electrically microstimulating somatosensory cortex. We found that using state-dependent algorithms that tracked the dynamics of ongoing activity led to an increase in the amount of information extracted form neural activity by 22%, with a consequently increase in all of the indices measuring the BMI's performance in controlling the dynamical system. This suggests that state-dependent decoding algorithms may be used to enhance BMIs at moderate computational cost.

## Introduction

The last two decades have seen tremendous progress in the development of brain-machine interfaces, or BMIs (Lebedev and Nicolelis, 2006; Leuthardt et al., 2009; Andersen et al., 2014; Bensmaia and Miller, 2014; Moxon and Foffani, 2015). These interfaces mediate communication between a brain and the external world, and hold an enormous potential for clinical applications. In particular, interfaces that decode neural activity (for example, decode the motor intent of the subject from premotor cortical activity), and then use this information to command an artificial actuator (for example, a robotic arm, a motorized wheelchair, or a computer cursor), can have a considerable clinical impact for the treatment of patients with neurological diseases such as stroke, spinal cord injury, or Parkinson's disease (Hochberg et al., 2012; Bouton et al., 2016; Capogrosso et al., 2016; Moraud et al., 2016).

The advances in BMIs have been assisted by technological progress that has increased the quality and quantity of the signals recorded from the brain and has improved current brain stimulation techniques (Wolpaw et al., 2000; Lebedev and Nicolelis, 2006; Brunner et al., 2011; Calixto et al., 2013; Tabot et al., 2013; Angotzi et al., 2014; Lebedev M., 2014; Wander and Rao, 2014; Gupta et al., 2016). Despite all these progresses, several aspects of BMIs remain to be addressed. One key problem is that the large trial-to-trial variability of neural responses (Faisal et al., 2008; Quian Quiroga and Panzeri, 2009) still strongly limits the performance of BMIs (Baranauskas, 2014; Lebedev M. A., 2014). This problem has proved difficult to overcome by simply increasing the number of recording or stimulating electrodes, because trial-to-trial variability of neural activity is largely shared among neurons. This shared variability partly arises from intrinsic network-level factors (which can be collectively described as “network state”) that include fluctuations in ongoing spontaneous activity and network excitability, and the level of neuromodulation as well as general changes in behavioral state such as the level of arousal (Goris et al., 2014; Lin et al., 2015; Schölvinck et al., 2015). This shared source of variability within a network can be conceptualized by thinking of neural activity as state-dependent: neural activity does not depend only on external task-related variables but also on internal endogenous network state variables, that are often collectively termed cortical state (Buonomano and Maass, 2009; Harris and Thiele, 2011; Ritter et al., 2015; Safaai et al., 2015).

We have recently proposed (Panzeri et al., 2016) that the problem of shared trial-to-trial variability of neural activity may in principle be addressed, or at least alleviated, by using decoding algorithms based on decoding rules that properly consider the state-dependence of neural responses and so are not confounded by the state-induced trial-to-trial variability of neural responses to stimuli. The simplest way to take advantage of state dependent rules is, assuming that state-induced variability is additive, to first estimate the amount of single-trial firing due to state-induced variability and to discount it by subtracting it out (Safaai et al., 2015; Panzeri et al., 2016). Although this suggestion has not been tested yet in BMIs, studies that have used models of state dependence of neural responses based on the observation of neural activity encourage us to think that this idea may work. In fact, studies using dynamical systems models of the time course of spontaneous activity has shown that it is possible to use such model of state dependence to obtain excellent predictions of each single trial responses to stimuli (Curto et al., 2009; Safaai et al., 2015). Importantly, one study (Safaai et al., 2015) has shown that good predictions of single trial responses can be used to dramatically improve (by up to ~70%) the performance of single trial decoding of stimulus information from neural activity.

Building on these recent results, here we put to test our proposal, by implementing state-dependent decoding rules in a motor BMI that controlled the movement of a simulated point mass moving into a viscous medium over a horizontal plane. In this BMI, we fed to the brain information about the position of the mass by electrically microstimulating somatosensory cortex of anesthetized rats. We then recorded neural activity from motor cortex in response to this electrical stimulation, and we decoded this motor activity to drive the point mass toward a target, until the target was reached. We compared the BMI performance obtained with state-independent decoding algorithms, that only used neural activity recorded in motor cortex after the brain received information about the mass position by microstimulation, with the performance of state-dependent decoding algorithms that also used ongoing motor cortical activity prior to stimulation to discount the state-induced variability of motor cortical activity in response to the information about the mass position. The quantitative comparison of the BMI performance with state-independent and state-dependent algorithms is useful to provide an initial evaluation of the possible advantages of using state-dependent BMIs.

## Materials and methods

### General scheme of the state-dependent BMI

**Figure 1:**
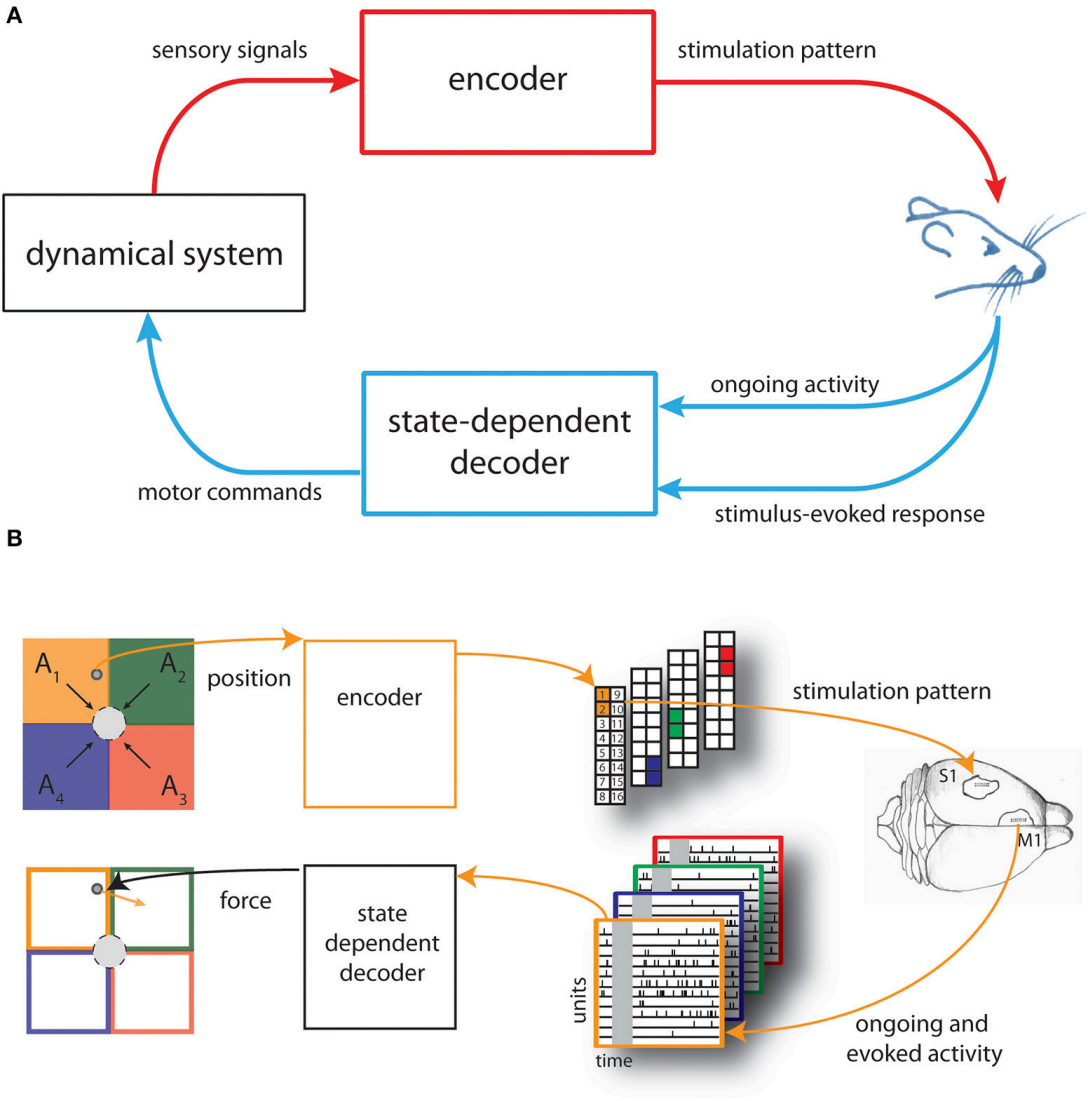
**Schematic of the state-dependent bidirectional brain-machine interface. (A)** We developed a state-dependent bidirectional BMI by connecting the brain of an anesthetized rat to a simulated dynamical system represented by a point mass moving in a viscous medium. An encoder translated sensory signals generated from the interaction of the dynamical system with the environment into brain stimulation patterns and a state-dependent decoder transformed the recorded neural activity—both the post-stimulus evoked and pre-stimulus ongoing cortical activity—into motor commands to control the external system. **(B)** The space in which the point mass can move was divided into four sensory regions (*A*_*1*_, …, *A*_*4*_) and four force vectors (*F*_*1*_, …, *F*_*4*_) (black arrows), pointing toward the target (gray circle), were placed in the centroid of each region. The actual position of the point mass was converted by the encoder into a pattern of intracortical microstimulation delivered to the somatosensory cortex (top from left to right). A state-dependent decoder transformed the neural activity recorded from the motor cortex of rat's brain into a force vector (orange arrow) to be applied to the point mass (bottom from right to left).

In this work we developed a novel neural interface, which we termed state-dependent BMI, by including a state-dependent decoder module to the dynamic Neural Interface described the first time in Vato et al. (2012) and then updated in Szymanski et al. (2011), Vato et al. (2014), Boi et al. (2015a), see Figure 1A. Here our main goal is to test the potential advantages of introducing such state-dependence decoding. The BMI is summarized briefly in what follows.

The main purpose of the BMI was to control the movement of a dynamical system (a simulated point mass moving in a viscous medium, for details see Text S1) to reach a positional target. Here we set-up the neural interface by dividing the space in which the mass can move, a square box, into four equal regions (*A*_*1*_,…,*A*_*4*_). We also defined four force vectors (*F*_*1*_,…,*F*_*4*_) placed in the centroid of each regions and pointing toward the target represented by a circular region placed in the center of the workspace (Figure 1B, top left).

Before each experimental session, we also chose four different couples of adjacent electrodes of a stimulating multielectrode arrays placed in the somatosensory cortex (S1) of an anesthetized rat.

An encoder provided the brain with the information about the position of the point mass by using these couples of electrodes to deliver different patterns of intracortical microstimulation (ICMS) denoted as *s*_*1*_, …, *s*_*4*_, each one associated to a single sensory region (Figure 1B, top-right).

Neural signals evoked by the micro-stimulation were recorded from the motor cortex (M1) by means of a recording multielectrode array and used to control the movement of the dynamical system. A decoder transformed the recorded signals into a force vector to be applied to the simulated point mass that moved according to its dynamics, for a predefined amount of time (Figure 1B, bottom from right to left).

The BMI task consisted in driving the simulated point mass toward a target region (a circle around the origin of variable size, see Results) by extracting from each stimulus-evoked neural response a force vector (*F*_*decoded*_) calculated as a weighted sum of the four force vectors (*F*_*1*_,…,*F*_*4*_).

### Neurophysiological procedures

All the neurophysiological procedures have been performed in accordance with DL 116/92 of the Italian legal code and approved by the institutional review board of the University of Ferrara and by the Italian Ministry of Health (73/2008-B). The procedures are identical to those reported previously and are only briefly summarized in what follows. We refer to Vato et al. (2012) for more details.

Neural data were recorded from five male Long-Evans rats (300–400 g) anesthetized for the entire duration of the experimental sessions by means of Xylazine (5 mg/kg) and a mixture of Tiletamine and Zolazepam (30 mg/kg) as an alternative of using narcotic drugs (Vogler, 2006). Two craniotomies were performed above the vibrissal representation of the primary somatosensory (S1) cortex and above the vibrissal motor cortex (M1) on the same hemisphere.

The stimulation microwire array (Tucker Davis Technologies—TDT) was lowered perpendicular to S1, 900–1100 μm under the surface (AP −3.5 mm, LM +4 mm with respect to the most posterior medial electrode of the array) using a hydraulic microdrive. Each stimulation pattern consisted of a train of 10 biphasic pulses (333 Hz pulse frequency, 200 μs pulse duration, 100 μA pulse amplitude, 30 ms train duration) delivered from 50 to 200 times during an experimental session.

The recording microwire array was placed at depth 300–500 μm below the pia (AP +1.5 mm, LM +0.5 mm with respect to the most posterior medial electrode of the array) using a hydraulic microdrive. These locations have been chosen for the presence of several cortico-cortical connections between the two regions (Ferezou et al., 2007; Chakrabarti et al., 2008; Mao et al., 2011). Both arrays had 16 microelectrodes (2 rows of 8 electrodes, 50 μm diameter) each one separated from the neighboring ones by 250 and 375 μm along and across the rows, respectively. After the insertion of the arrays, we let the electrodes to settle down for 30 min and then we run a commercial available spike-sorting software (Rasputin, Plexon Inc.) identifying 10–15 single units per experimental session that were active from the beginning to the end of each experimental session which could last from 20 to 60 min. To get an estimate of the order of magnitude of the stability of the responses over the experimental session, we evaluated for each unit the variation in the duration of the whole experimental session of the stimulus-evoked activity. By averaging the linear slope across all the units, across all the stimuli and across all the experimental sessions, we found an average linear regression slope of β = 0.22 ± 0.18 mHz/trial and a percentage variation of +20.5 ± 16.35% with respect to mean firing rate. This moderate drift of evoked firing rate over the session is one of the sources of state dependency of neural responses that could be discounted by state dependent algorithms.

An illustration of the kind of recordings and responses that we collected in this way is reported in the bottom-right of Figure 1B, where we show the raster plots of spike trains during the pre- and post-stimulus window of single-units recorded in motor cortex.

### State-dependent decoder

#### Terminology and assessment of cortical states

**Figure 2:**
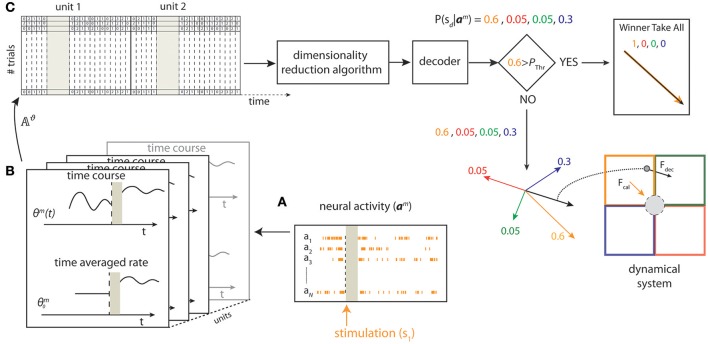
**Schematic of state-dependent decoding algorithm**. We used a stimulating array placed in the somatosensory cortex to deliver one out of four different patterns of intracortical microstimulation (*s*_*1*_, *s*_*2*_, *s*_*3*_, *s*_*4*_) according to the position of the point mass. **(A)** The pre-stimulus ongoing activity and the stimulus-evoked responses (***a***_1_, …, ***a***_*N*_) were collected from a 16-channels recording array placed in the motor cortex. **(B)** For each trial *m*, we defined the state Θ^*m*^ from the pre-stimulus ongoing activity based on the time averaged pre-stimulus activity or by considering the time course using temporal bins of 5 ms. **(C)** After a spike-sorting algorithm discarded the stimulus artifacts (gray area), both the state variables and the post-stimulus responses were stored in a matrix (𝔸^ϑ^). For each trial *m*, a Principal Component Analysis (PCA) algorithm reduced the dimensionality of the matrix and a decoder then provided a probability $P\left(s_{d}|{\text{a}}^{m}\right)$ representing the posterior belief that stimulus *s*_*d*_ was presented after observing activity ***a***^*m*^. To improve the BMI performance, the largest value among the four probabilities was compared to a predefined threshold (*P*_*thr*_) to select either a *Winner Take All* or a *Weighted Sum* procedure to obtain the direction and the magnitude of the decoded force, *F*_*decoded*_, to be applied to the point mass (black arrow bottom right) from the four force vectors (*F*_*1*_,…,*F*_*4*_) (colored arrows).

The scheme of the state-dependent decoding algorithm is shown in Figure 2. We considered the ongoing activity in a pre-stimulus time window of duration Δ*T*_*pre*_*stim*_ and the stimulus-evoked response in a post-stimulus time window of duration Δ*T*_*post*_*stim*_. We recorded the evoked and the ongoing Single Unit Activity (SUA), for each unit *n* ∈ {1, …, *N*}, where *N* is the total number of units for each experimental session. We discretized the SUA by binning neural spike trains into short time intervals of 5 ms and then computing the number of spikes in each interval. As a first preliminary approach, we used uniform time binning of shortest size (5 ms) both for evoked response and for pre-stimulus ongoing activity. However, when developing the preliminary analyses, we noted that considering the ongoing activity immediately before the stimulus onset with fine (5 ms) temporal precision allowed a better performance in state-dependent decoding, whereas the beneficial effect of decoding when considering pre-stimulus activity recorded further back from the stimulus onset window did not need recording spikes with such millisecond-scale temporal precision (for details see Text S2). For this reason, we used for the pre-stimulus window variable time bin sizes (finer bins closer to stimulus onset and coarser bins further from stimulus onset) using a total of 39 time bins organized as shown in Table S1. Adaptive-size time bins gave an information advantage of ~7% with respect to using fixed-shortest-size bins.

We denote the neural spike count as **R**^*m*^ for the evoked response, and as **Θ**^*m*^ for the ongoing activity, for each trial *m* ∈ {1, …, *M*}, where *M* is the total number of trials. Each row of **R**^*m*^ and **Θ**^*m*^ corresponds to one unit and each column corresponds to one time-bin *t*. For each trial *m*, we denote as **r**^*m*^(*t*) and **θ**^*m*^(*t*) the columns of **R**^*m*^ and **Θ**^*m*^, respectively. The vectors **r**^*m*^(*t*) and **θ**^*m*^(*t*) represent the population spike counts in bin *t* for the evoked response considered in the time window of duration Δ*T*_*post*_*stim*_ and for the pre-stimulus ongoing activity considered in the time window of duration Δ*T*_*pre*_*stim*_, respectively. We have ${\text{R}}^{m}\in \;{\mathbb{R}}^{NxT_{r}}$, with *T*_*r*_ being the number of time bins per trial considered in the post-stimulus window, and $\Theta^{m}\in \;{\mathbb{R}}^{NxT_{\theta }}$, with *T*_θ_ being the number of time bins per trial considered in the pre-stimulus window. Note that *T*_*r*_ and *T*_θ_ depend on Δ*T*_*post*_*stim*_ and Δ*T*_*pre*_*stim*_ respectively and are constant across all trials. The time bin immediately after the stimulation was labeled as bin #1 (*t* = 1) and the last time bin of the post-stimulus time window was labeled as bin #*T*_*r*_ (*t* = *T*_*r*_). The time bin immediately before the stimulation was labeled as bin #–1 (*t* = −1) and the first time bin of the pre-stimulus time window was labeled as bin #–*T*_θ_ (*t* = −*T*_θ_).

For each trial *m*, we defined the Single Unit Activity (SUA) state variables matrix as:

(1) $$\begin{array}{l} \Theta^{\text{SUA},m}=\left[\theta^{m}\left(-T_{\theta }\right),\;\ldots ,\;\theta^{m}\left(-t\right),\ldots ,\theta^{m}\left(-1\right)\right]\in {\mathbb{R}}^{N\text{x}T_{\theta }} \end{array}$$

For each trial *m* we defined the SUA state activity matrix as:

(2) $$\begin{array}{l} A^{\text{SUA},m}=\left\{\Theta^{\text{SUA},m}|R^{m}\right\}\\ \;=\{\left[\theta^{m}\left(-T_{\theta }\right),\;\ldots ,\;\theta^{m}\left(-t\right),\ldots ,\theta^{m}(-1)\right]\\ \;|\text{ ​}\left[r^{m}\left(1\right),\text{​ }\ldots ,\text{​ }r^{m}\left(t\right),\text{​}\ldots ,\text{​}r^{m}(T_{r})\right]\}\in {\mathbb{R}}^{N\text{x}\left(T_{\theta }\;+\;T_{r}\right)}\; \end{array}$$

i.e., we obtained **A**^*SUA,m*^ by concatenating the SUA state variable matrix and the response matrix (**see** Figure 2, top left). In some sessions, we found that there were fewer single units and there was little difference in the response profiles of different single units to the four stimuli. In such case, it was more convenient, faster and robust to decode neural activity after neglecting single unit identity and pooling together the activity of all units into a single Multi Unit Activity (MUA) channel. In such sessions, we defined a MUA state variables matrix and a MUA state activity matrix to describe the network state exactly as we did for the SUA case above (for details see Text S3). The quantitative criterion to choose between using SUA or MUA was to select the signal that optimize the decoding performance as described in Section Decoding Algorithm.

We denoted as **A**^ϑ,*m*^ the generic state activity matrix where ϑ could be one of SUA or MUA.

For each trial *m*, the rows of **A**^ϑ,*m*^ correspond to the units and the columns to time bins, i.e., $a_{n,t}^{\vartheta ,m}\in {\text{A}}^{\vartheta ,m}$ is the spike count at time *t* recorded from unit *n*. We reshaped **A**^ϑ,*m*^ in a row vector **a**^ϑ,*m*^ by concatenating all of its rows:

(3) $$\begin{array}{l} {\text{a}}^{\vartheta ,m}=\left[a_{1,-T_{\theta }}^{\vartheta ,m},\ldots ,a_{1,-1}^{\vartheta ,m},a_{1,1}^{m},\ldots ,a_{1,T_{r}}^{m},\ldots ,a_{N,-T_{\theta }}^{\vartheta ,m},\ldots ,a_{N,-1}^{\vartheta ,m},a_{N,1}^{\vartheta ,m},\ldots ,a_{N,T_{r}}^{m}\right]\in {\mathbb{R}}^{N\left(T_{\theta }\;+\;T_{r}\right)} \end{array}$$

We called **a**^ϑ,*m*^ state-dependent activity vector.

For each trial *m*, given a stimulus *s*_*m*_ ∈ {*s*_1_, *s*_2_, *s*_3_, *s*_4_} and a corresponding activity vector **a**^ϑ,*m*^, we defined as decoder the function ${ŝ}_{m}=\text{D}\left({\text{a}}^{\vartheta ,m}\right)$, where ŝ_*m*_ denotes the predicted stimulus.

To test the importance of taking into account the network state and also the usefulness of defining state variables in terms of the temporal history, rather than only of the strength of the pre-stimulus activity, we performed three different decoding operations. In each of the three types of decoding the mathematical algorithm was the same, but what varied was the neural activity that we fed to the decoder.

In the first case, that we called state-independent decoder (shortened to SI decoder), we fed to the decoder an activity matrix that did not contain the pre-stimulus activity (in other words, we fed to the decoder only the response matrix). This decoder did not use state dependence. In this case ϑ = *SI* and **a**^ϑ,*m*^ = ***r***^*m*^, where ***r***^*m*^ denotes the response vector (or state-independent activity vector).

In the second case, that we called state-dependent time-dependent decoder (shortened to SD-TD decoder), we fed to the decoder an activity matrix that did contain both the response matrix and also the full temporal representation of the pre-stimulus activity, in Equation (4). This decoder took advantage of knowledge of network state, and used the temporal structure of ongoing activity to determine state.

In the third case, that we called state-dependent time-averaged decoder (shortened to SD-TA decoder), we fed to the decoder an activity matrix that did contain both the response matrix and also the time-averaged pre-stimulus activity, in Equation (4). This decoder took advantage of knowledge of network state, but used only the strength of pre-stimulus activity, and not its time course, to determine state.

In all calculations we did, we fed to the decoder an activity matrix that contained spike timing information in the response (post-stimulus) period. This is because this operation maximized the amount of state-dependent information, and this made it more difficult for the state dependent decoder to add information. This ensured that our results of state dependent information increase did not result from suboptimal consideration of the temporal structure of the responses to the stimuli.

The state variables defined above are defined and computed in the time domain. To check if a more compact and informative definition of state could be obtained in the frequency domain, we did the following control analysis. Neural oscillations are usually quantified using LFPs. However, we could record LFPs only in one session out of 13. Thus, we could not relate systematically the pre-stimulus LFP oscillations to the state variations. Given this, and to understand whether state variables could be described with simple oscillatory parameters, in all sessions we first built the overall session MUA by pooling the spikes of all neurons and we then convolved it with a Volterra kernel (Poor, 1988) to generate an analog population signal (termed convolved spiking activity in the following) that is similar to the LFP (the Volterra kernel was computed from the only session in which we could record both LFPs and spikes, and was similar in width and shape to those reported in other studies; Kreiman et al., 2000; Rasch et al., 2009). This process has been reported to generate a convolved MUA signal that correlates well with the LFP recorded from the same location (Kreiman et al., 2000; Rasch et al., 2009). We checked this match in the session where we had both spiking activity and LFP and we observed a good correlation between the convolved MUA signal and the LFP (ρ = 0.61, *p* < 0.001). We then filtered the convolved MUA signal in the (1–4 Hz) band and we quantified the gain in state-dependent information that we obtained using either phase or power of this filtered convolved MUA signal. We found that the information gain obtained using this definition of state was 6.12 ± 1.21%, thus smaller than that obtained with the timed-domain state computation presented throughout this paper. Information gains obtained when filtering this signal in frequency bands of higher frequency were even smaller. We thus decided to use throughout the paper the time-domain state computation procedure described above.

#### Decoding algorithm

Taking into account all trials *m* ∈ {1, …, *M*}, we built the state-dependent activity matrix 𝔸^ϑ^ by shaping all the *M* vectors **a**^ϑ,*m*^ in rows:

(4) $$\begin{array}{l} {𝔸}^{\vartheta }=\left[\begin{array}{c} {\text{a}}^{\vartheta ,1}\\ \vdots \\ {\text{a}}^{\vartheta ,M} \end{array}\right]=\left[\begin{array}{ccc} a_{1,-T_{\theta }}^{\vartheta ,1} & \cdots & a_{N,T_{r}}^{1}\\ \vdots & \ddots & \vdots \\ a_{1,-T_{\theta }}^{\vartheta ,M} & \cdots & a_{N,T_{r}}^{M} \end{array}\right]\in {\mathbb{R}}^{M\text{x}N\left(T_{\theta }\;+\;T_{r}\right)} \end{array}$$

For each row of 𝔸^ϑ^ we had *N*(*T*_θ_ + *T*_*r*_) trial-independent variables.

We used multiclass Linear Discriminant Analysis (LDA) (Rao, 1948; Delis et al., 2016) to predict the stimulus associated with single trial activities using a leave-one-out cross-validation. We denote by ${𝔸}_{tr}^{\vartheta }\in {\mathbb{R}}^{\left(M-1\right)\text{x}N\left(T_{\theta }+T_{r}\right)}$ the activity matrix corresponding to the training set and by 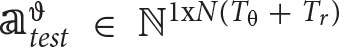 the activity vector corresponding to the test trial. Before training the LDA decoder we reduced the dimensionality of ${𝔸}_{tr}^{\vartheta }$ by Principal Components Analysis (PCA) decomposition (Jolliffe, 2002; Onken et al., 2016), and selecting the *k* principal components of ${𝔸}_{tr}^{\vartheta }$ (see algorithm step-by-step description in Text S4):

(5) $$\begin{array}{l} {𝔸}_{tr}^{\vartheta }={\text{H}}_{tr}^{\vartheta ,k}{\text{B}}_{tr}^{\vartheta ,k}+\text{residual} \end{array}$$

where ${\text{H}}_{tr}^{\vartheta ,k}\in {\mathbb{R}}^{\left(M-1\right)\text{x}k}$ denotes the principal component scores matrix and ${\text{B}}_{tr}^{\vartheta ,k}\in {\mathbb{R}}^{k\text{xN}\left(T_{\theta }+T_{r}\right)}\;$ the principal components loadings matrix. We trained the decoder on ${\text{H}}_{tr}^{\vartheta ,k}$ with presented stimulus indices *s*_*i*_ as class labels and we tested the decoder on 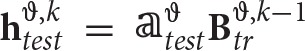.

For each test trial *m* and each *d* ∈ {1, 2, 3, 4}, the decoder then provided a probability $\text{P}\left(s_{d}|{\text{a}}^{\vartheta ,m}\right)$ representing the posterior belief that stimulus *s*_*d*_ was presented after observing activity **a**^ϑ,*m*^. To improve decoding performance, we further adopted a Winner Take All (WTA) strategy to improve the decoding performance (Maass, 2000) if, for a given trial *m*, the largest of the probabilities $\text{P}\left(s_{1}|{\text{a}}^{\vartheta ,m}\right),\ldots ,\text{P}\left(s_{4}|{\text{a}}^{\vartheta ,m}\right)$ was greater that a certain threshold *P*_*thr*_:

(6) $$\begin{array}{l} \text{if }\underset{d}{\max}\left(\text{ P}\left(s_{d}|a^{\vartheta ,m}\right)\right)\leq P_{thr}\\ \;\tilde{\text{P}}\left(s_{d}|a^{\vartheta ,m}\right)=\text{ P}\left(s_{d}|a^{\vartheta ,m}\right)\\ \text{if }\underset{d}{\max}\left(\text{ P}\left(s_{d}|a^{\vartheta ,m}\right)\right)>P_{thr}\\ \;\{\begin{array}{ll} \tilde{\text{P}}\left(s_{d}|a^{\vartheta ,m}\right)=1 & \text{if }d=\underset{d}{\text{argmax}}\left(\text{ P}\left(s_{d}|a^{\vartheta ,m}\right)\right)\\ \tilde{\text{P}}\left(s_{d}|a^{\vartheta ,m}\right)=0 & \text{otherwise} \end{array} \end{array}$$

For all test trials, we stored these probabilities in a matrix $\tilde{\text{P}}\in {\left[0,1\right]}^{M\text{x}4}$, where each element ${\tilde{\text{p}}}_{d,m}≜\tilde{\text{P}}\left(s_{d}|{\text{a}}^{\vartheta ,m}\right)$.

#### Decoding performance measure

To quantify decoding performance, we used an information theoretic characterization (Shannon, 1948; Quian Quiroga and Panzeri, 2009).

In particular, we first computed the confusion matrix **Q** ∈ [0, 1]^4x4^ quantifying the probability that a presented stimulus *s*_*i*_ is decoded as stimulus *s*_*d*_. Each element **Q** is defined as follows:

(7) $$\begin{array}{l} {\text{q}}_{d,i}=Q\left(s_{d}|s_{i}\right)≜\;\sum_{m:\;s_{m}=s_{i}}\tilde{\text{P}}\left(s_{d}|{\text{a}}^{\vartheta ,m}\right)\text{P}\left(s_{m}=s_{i}\right) \end{array}$$

We then quantified decoding performance as the mutual information in the confusion matrix:

(8) $$\begin{array}{l} I\left(S;D\right)=\sum_{d,i}\text{P}\left(s_{i}\right)Q\left(s_{d}|s_{i}\right)\log_{2}\frac{Q\left(s_{d}|s_{i}\right)}{Q\left(s_{d}\right)} \end{array}$$

where *P*(*s*_*i*_) is the probability that electrical stimulus *s*_*i*_ was applied, and *Q*(*s*_*d*_) is the marginal probability of the decoded stimulus in the confusion matrix (Shannon, 1948).

Information in the confusion matrix is measured in bits (1 bit corresponds to a reduction of uncertainty by a factor of two). It quantifies the amount of information can be extracted by decoding the stimulus from neural activity with a given decoding algorithm. In our case, information quantities obtained from the SI, SD-TA, and SD-TD algorithms quantify the information that can be extracted from neural responses without knowledge of network state, with the knowledge of the temporal structure of the network state prior to the stimulus, and with the knowledge of the time-averaged strength of the network state prior to the stimulus.

A systematic error in this measure of information was introduced by the fact that the stimulus conditional responses probabilities were calculated over a finite number of trials (Panzeri et al., 2007). We corrected this bias by using a shuffling procedure (Montemurro et al., 2007).

#### Decoder parameters optimization

We used the information in the confusion matrix *I*(*S*; *D*) to optimize the parameters *P*_*thr*_, *k*, ϑ, *T*_θ_ and *T*_*r*_. We chose the parameter values that maximized *I*(*S*; *D*), by using leave-one-out cross-validation on the *M*-1 trials of the training set. We then used the optimized parameters to evaluate the test trial (more details are presented in Text S4 and Figure S1).

#### Decoded force vector calculation

For each trial *m*, the decoded force vector was computed as weighted sum of the four forces (*F*_1_,…,*F*_4_*)* defined during the set-up of the neural interface using $\tilde{P}\left(s_{d}|{\text{a}}^{\vartheta ,m}\right)$ as weights (see Figure 2)

(9) $$\begin{array}{l} {\vec{F}}_{decoded}^{m}\;=\;\sum_{d=1}^{4}\tilde{\text{P}}\left(s_{d}|{\text{a}}^{\vartheta ,m}\right){\vec{F}}_{d}\; \end{array}$$

## Results

Neural responses to a sensory stimulation depend not just on the sensory input but also on the intrinsic network variables defined as network state (Buonomano and Maass, 2009). In this paper we tested the hypothesis that the implementation of a decoding algorithm that take into account state-induced variability in the neural responses may increase the performance of BMIs (Figures 1, 2).

We used state-dependent decoding rules in a motor BMI that controlled a simulated dynamical system represented by a point mass moving in a viscous medium (Vato et al., 2012). Information about the position of the point mass was fed to the brain of an anesthetized rat by electrically microstimulating the somatosensory cortex, and the neural activity recorded from motor cortex was decoded and used to drive the mass toward a target, until the target was reached. The space in which the mass could move was divided into four contiguous sensory regions each of them associated with a different pattern of intracortical stimulation (*s*_1_, *s*_2_, *s*_3_, *s*_4_) (Figure 1B). The original, state-independent, implementation of this BMI (Vato et al., 2012) was inspired by the spinal cord, which mediates movements by generating positions-dependent force fields (Bizzi et al., 1991; Mussa-Ivaldi et al., 1994; Tresch and Bizzi, 1999). Here, this BMI was used because our extensive experience with it allowed us to be confident to run it optimally in the state-independent decoding case, which insured that the gain in performance observed with state-dependent rules was not due to fact that the stat-independent ones were inefficiently implemented.

We compared the BMI performance obtained by using a state-independent decoder with the performance obtained by using decoder algorithms that take into account the dependence of neural responses on the cortical state (Figure 2).

The term cortical state is used in the literature to describe a wide range of phenomena (Curto et al., 2009; Harris and Thiele, 2011; Marguet and Harris, 2011; Pachitariu et al., 2015). These phenomena range to the fluctuations of ongoing activity prior to stimulation reflecting endogenous changes in the network's excitability (Azouz and Gray, 1999; Lakatos et al., 2005; Curto et al., 2009; Kayser et al., 2015). Other definition of state include the history of stimulation prior to the current stimulus (Buonomano and Maass, 2009), fluctuations in attention (Harris and Thiele, 2011) and/or arousal that correlate also with biomarkers such as pupil state (Costa and Rudebeck, 2016; Joshi et al., 2016), and fluctuations in neuromodulation (Safaai et al., 2015). Here we only consider as state variables those corresponding to ongoing fluctuations of the network's excitability and quantifiable through examining ongoing network activity a few hundreds of milliseconds prior to the application of the stimulus.

To examine the usefulness of knowing network state (either through the detailed examination of the time course of pre-stimulus activity or through the simpler examination of only the time-averaged pre-stimulus activity) we run the BMI with three different decoders. The first, that we called state-independent decoder (shortened to SI decoder), did not use the pre-stimulus activity but only used the post-stimulus responses. The second, that we called state-dependent time-dependent decoder (shortened to SD-TD decoder), used both the post-stimulus responses and the full temporal representation of the pre-stimulus activity. The third, that we called state-dependent time-averaged decoder (shortened to SD-TA decoder), operated on post-stimulus responses and on the time-averaged pre-stimulus activity.

To summarize, the SI decoder did not use state dependence at all. Of the two state dependent decoders, one (SD-TA) used only the strength of pre-stimulus activity to determine the network state, whereas the SD-TD used also the pre-stimulus activity time course to determine state.

### Decoding performance

We first studied the decoding performance of the BMI when run with these three different decoding algorithms.

**Figure 3:**
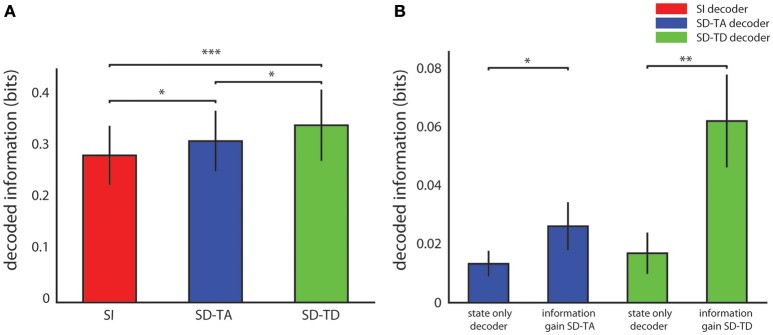
**Decoding performance of state-dependent and state independent algorithms. (A)** Information about the stimulus delivered in the somatosensory cortex, carried by the neural activity recorded in the motor area. We compared the state-independent decoder (SI, red bar) with two different state-dependent decoders: the state-dependent time average decoder (SD-TA), which uses state but only use time averaged pre-stimulus activity to define state), and the state-dependent time-dependent decoder (SD-TD), which uses state and uses the full timing of pre-stimulus activity to define state). **(B)** Comparison between the information gain due to the introduction of the state variable(s), computed as the difference between the information decoded from knowledge of state (either SD-TD, green bars; or SD-TA, blue bars) and the decoded information that the state variable carried about the stimulus. The bars report the average ± S.E.M of the information over all experimental sessions. In all figures, ^*^, ^**^, and ^***^ denote significance values of *p* < 0.05, 0.01, and 0.001, respectively (two-sided Wilcoxon signed rank test).

Figure 3A reports the average over all experimental sessions of the information decoded with each algorithm. The SD-TA decoder, that took into account the state but ignored its time structure, extracted 0.026 ± 0.008 bits of information, a larger information amount (*p* = 0.0015, two-sided Wilcoxon signed rank test) than the state-independent decoder. The SD-TD decoder that took into account state and used in full its time structure extracted 0.062 ± 0.016 bits, a significantly larger amount of information (*p* = 2.44e-4, two-sided Wilcoxon signed rank test) with respect to both the SI decoder and the SD-TA decoder (*p* = 0.0098, two-sided Wilcoxon signed rank test). The percentage information gain with state dependent decoders over the state-independent decoder was 9.68% for the SD-TA and 21.74% for the SD-TD decoder, respectively.

We then asked whether the gain in information when taking into account state dependence could be explained because the state variables itself was informative about the stimulus. A direct relationship between stimulus and state can for example arise when the same stimulus, applied for several consecutive trials, creates a bias (due to habituation) in the pre-stimulus ongoing activity. To address this issue, we calculated the amount of information that the state variable (either defined with or without taking into account the temporal structure of ongoing activity) carried about the stimulus. This value was calculated using similar decoding procedures as for the other information quantities, but not feeding the post-stimulus response to the decoder. We found that the information carried by both state variables about the stimulus was significantly smaller (a factor of two for the SD-TA gain, *p* = 0.047, and a factor of 4 for the SD-TD gain, *p* = 2.44e-4) than the information gain of the state dependent decoders (see Figure 3B). These results show that the information gain of both the state dependent decoders was not merely a consequence of the fact that the state variable carried some stimulus information, but the information gain was in its majority due to the fact that the knowledge that response to the stimulus depends on the state helps the decoder.

**Figure 4:**
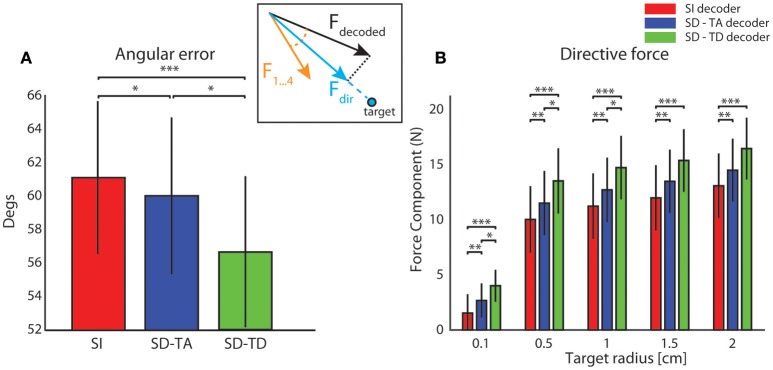
**Magnitude and direction of the decoded force vectors. (A)** The angular error is defined as the angle (degrees) between the force vector (*F*_*decoded*_, black arrow) extracted by the decoder from the recorded signal and one of the four force vectors (*F*_*1*_,…,*F*_*4*_ orange arrow) associated to the sensory region to whom the actual position of the point mass belongs and. The graph shows the average ± S.E.M of the angular errors of the decoded forces over all experimental sessions by comparing the state-independent vs. both the state-dependent decoding algorithms (SD-TA and SD-TD). The angular error gives an estimation of the decoder performance independently of the trajectories generated by the dynamical system. **(B)** The directive force is defined as the magnitude of the component of the decoded force pointed toward the target (*F*_dir_, cyan arrow in the inset). The graph shows, in contrast with the angular error, how the average magnitude angular error of the directive forces depends on the target radius. For each values of the target radius (from 0.1 to 2 cm) we collected 800 trajectories and we compared the average (± S.E.M) values of the magnitude of such directive force using state-independent (red bars) and state-dependent decoders (the blue bars for the SD-TA decoder and the green bars for the SD-TD decoder).

Figure 4A reports the average value, over all experimental sessions, of the angle between the force produced by the decoding process (*F*_*decoded*_) and the expected force represented by one of the four force vectors (*F*_*1*_,…,*F*_*4*_) defined for each sensory region to set-up the neural interface (yellow arrow, Figure 4 inset). A perfect decoding would have led to an angle equal to zero, while any deviation from zero can been considered as a decoding angular error. Taking into account the temporal structure of the state, the SD-TD decoding algorithm led to a significant reduction of the angular error with respect to the SI decoder (−4.61 ± 1.15 degrees; *p* = 2.44e-4, two-sided Wilcoxon signed rank test). The SD-TA decoding algorithm led to a smaller, but significant, reduction of the angular error with respect to the SI decoder (−1.27 ± 0.74 degrees; *p* = 0.013, two-sided Wilcoxon signed rank test). The percentage reduction of angular error with state dependent decoders over the state-independent decoder was 2.07% for the SD-TA and 7.52% for the SD-TD decoder, respectively.

### BMI performance

**Figure 5:**
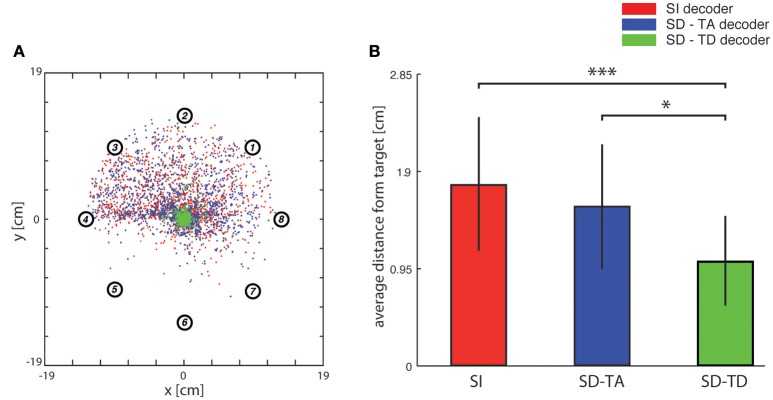
**Analysis of trajectories. (A)** In this graph red dots indicate, for each trajectory, the closest points to the target (i.e., the center of the horizontal plane) when the network state information is not considered. Blue and green dots represent the cases in which decoders take into account state variables from the ongoing neural activity. Data were collected by fixing the number of steps for each trajectory (i.e., 100) and by running the system 100 times for each of the eight predefined initial positions represented by circled numbers using the three different decoding algorithms. **(B)** Bar plots of the average distance of the closest points from the target of the trajectories generated by a state-independent decoder (red bars) and by state-dependent decoders (blue bar for the SD-TA decoder and green bar for the SD-TD decoder).

We then explored if and how an increase in decoding performance using a state-dependent algorithm was reflected into an increase in the BMI performance in terms of generated trajectories of the simulated dynamical system. The dynamical system controlled by the BMI was a simulated point mass placed in a simulated viscous medium and moving in a 36 × 36 cm simulated horizontal plane. The goal of the BMI was to move the point mass toward a target—a circular region placed in the center of the plane (Vato et al., 2012; Boi et al., 2015b, 2016) whose size was varied throughout the next subsections depending on the analysis to be performed. We initialized the position of the point mass at one of eight starting positions (circled numbers in Figure 5A). The point mass moved according to the sequence of decoded forces that the closed-loop BMI applied to the dynamical system in order to reach the target. For each experimental session we analyzed the decoded force vectors after collecting 100 trajectories for each starting positon. We measured the magnitude of the component of the decoded force pointing toward the target region, here referred to as Force Directed to the target (*F*_*dir*_, cyan arrow in Figure 4A inset). For each experimental session we collected 800 trajectories and we averaged across all the decoded forces to obtain the average *F*_*dir*_ force magnitude. We varied the target radius values from 0.1 to 2 cm repeating the calculation for all the 13 experimental sessions, for the five values of the target radius and for the three decoder (SI, SD-TA, and SD-TD) comparing the average *F*_*dir*_ force magnitudes in the three decoding conditions. We found a significant increase in the *F*_*dir*_ force magnitude when we considered the temporal structure of the state with respect to the SI decoding for all the target radius values; we had an average increase by 33.96% (average over the different target radius values). We had also an average increase by 14.68% in the *F*_*dir*_ force magnitude when we considered the time averaged pre-stimulus activity as state with respect to the SI decoding, for all the target radius values (Figure 4B).

#### Analysis of BMI trajectories

In order to evaluate the precision of the trajectories throughout the entire space in which the point mass could move we first run an analysis in which we set the target to a single point in the origin (thus the target region had a zero area and a zero radius). In this specific analysis, we stopped the running of the BMI after 100 time steps. We run the BMI as described in the previous section in order to explore whether the trajectories directed toward a point target (i.e., the origin of axes) got closer to the target when we used a state-dependent decoder with respect to a state-independent decoder. For each trajectory, we marked and plotted the positon of the point that was closest to the target point (the origin of the axes in the plane where the point mass moved). In Figure 5A we show an example of a collection of closest points to target obtained for one of the 13 experimental session used for this study (see Section Neurophysiological procedures). For each starting position, we repeated the experiment 100 times, yielding 800 points for each of the three decoders (red points for the SI decoder, blue points for the SD-TA decoder and green points for the SD-TD decoder). For each point shown in Figure 5A we measured the distance from the origin of the axes, we averaged these values and we obtained the average distance from the target. We repeated the calculation for all the experimental sessions and for the three decoders (SI, SD-TA, and SD-TD). We compared the average distances obtained by using each of the three decoders (Figure 5B). The closest points to the target extracted from the trajectories generated by running a state-dependent decoding algorithm were, on average, significantly closer to the target than using a state-independent decoder. Specifically, we found a significant decrease in the average distance from the origin of the axes of the closest points to target when the decoder considered the temporal structure of the state with respect to the SI decoder (−3.94 ± 1.00 mm, −35.54%; *p* = 4.88e-4, two-sided Wilcoxon signed rank test); we did not find a significant decrease when the decoder considered the time averaged pre-stimulus activity as state with respect to the SI decoder.

#### Convergence rate and speed increase of BMI trajectories

In order to evaluate how often and how fast the BMI can reach a target, we then run the BMI by setting a finite-size target region around the origin to be reached, and we tested the performance of the BMI in reaching target regions of different sizes (varying from a target area radius of 0.1 cm to a radius of 2 cm). We quantified this performance by computing both the rate of convergence of the BMI (that is, the percentage of trajectories generated by the BMI that converged to reach the target region) and the average number of steps taken to reach the target (the latter served to evaluate the speed by which the BMI reaches the target).

We defined the target as circular region with radius *r* placed in the center of the horizontal plane. We distinguished between converging and non-converging trajectories. A trial was considered successful (i.e., the trajectory in that trial was classified as converging) if the point mass reached within the border of the circular area. When this happened the BMI was stopped and we recorded the number of steps needed to reach the target. A trial was considered unsuccessful (non-converging trajectory) if after 100 steps the point mass did not reach the target region. We computed the performance of the BMI for each tested value of the target radius.

**Figure 6:**
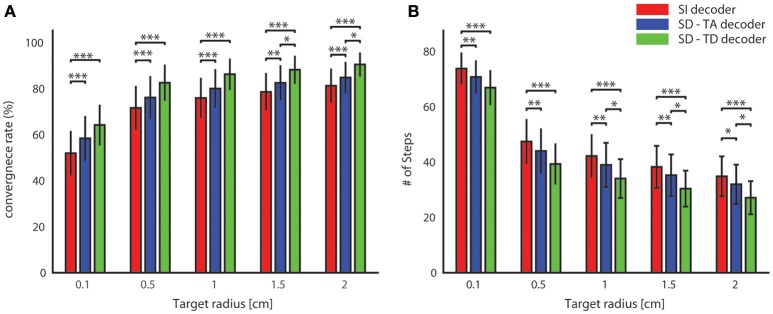
**Convergence rate and speed of the BMI**. The percentage of trajectories that reached the target and the length of these converging trajectories are described respectively by the average percentage of converging trajectories **(A)** and by the average number of steps **(B)** needed to reach the target. These two features define the performances of the bidirectional BMI and depend on the radius of the target. In these graphs we compared these two measures by running a state-independent (red bars) and two different state-dependent decoders (blue bar for the SD-TA decoder and green bar for the SD-TD decoder).

For all tested values of the target radius, we found (Figure 6) a significantly superior performance of the state-dependent decoders over the state independent decoder. The state dependent decoder that considered the temporal structure of pre-stimulus activity led to a better BMI performance than the one that did not consider the temporal structure of pre-stimulus activity. On average across all the radii of the target regions considered in Figure 6, use of the state-dependent decoder that considered the temporal structure of pre-stimulus activity increased the convergence rate by 10.49%, and it decreased the number of steps to reach the target by 16.38% with respect to the state-independent case. When we used a state-dependent decoder that used only the time-averaged pre-stimulus activity the convergence rate increased (on average across all sessions and tested target radius sizes reported in Figure 6) by 4.52% and the number of steps to reach the target decreased on average by 6.56% when compared to the state-independent case.

#### BMI trajectories stability and reproducibility

**Figure 7:**
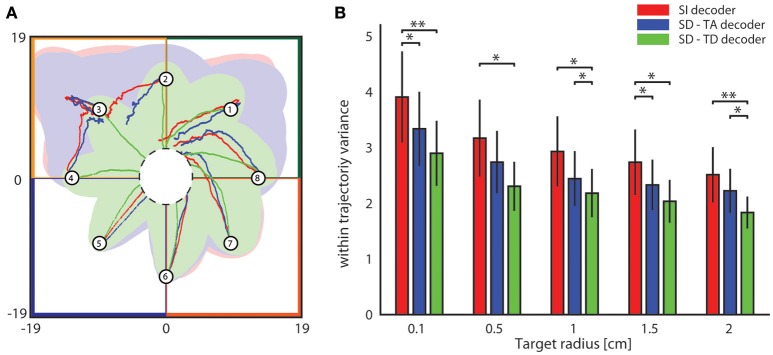
**Analysis of the dependence of the shape of trajectories on the decoding algorithms. (A)** The colored lines represent the mean trajectories and the shaded areas define the covariance of the trajectories generated by running the interface 100 times from each initial position (numbered circles). The state-independent decoding algorithm is represented by the red lines, the blue and the green lines represent the mean trajectory generated by using respectively the SD-TA and SD-TD decoders. The target region is represented by a circle placed in the origin of the workspace that is subdivided into four sensory regions represented with different colors. **(B)** The mean within-trajectory variance (*wtv* ± S.E.M) depends on the target radius and represents an index of the repeatability of the trajectories by using state-independent (red bars) and state-dependent decoders (blue and green bars).

We then investigated the stability and reproducibility of the trajectories obtained with state-dependent and state-independent decoders. We took the within trajectory variance, shortened to *wtv* and defined as $\sqrt{{C_{x}}^{2}+{C_{y}}^{2}}$ where *C*_*x*_ and *C*_*y*_ is the covariance of the distribution of per-step displacement along the *x* and *y* axis, respectively (Boi et al., 2016), as a measure of how the trial-to-trial variability is reflected into the shape of the generated trajectories. Results of how *wtv* varies when considering state-independent or state-dependent BMIs are reported in Figure 7. To compute these results, we initialized the position of the point mass at one of eight starting positions (circled numbers in Figure 7A) and, for each trajectory, we stopped the BMI when the point mass reached the target region or, if it did not reach it, at a maximum number of steps equal to 100. For each experimental session, we collected 100 trajectories for each starting positon. We considered the mean *wtv* obtained by averaging the *wtv* computed for each set of trajectories that started from one initial position. Figure 7A shows the mean trajectories (red lines) and the covariance (light red area) generated during one experimental session with the state-independent decoder. In this experimental session we set a target radius of 2 cm. In the correspondent SD-TA and SD-TD cases, the mean trajectories (blue and green lines, respectively) and the covariance (light blue and light green areas, respectively) are also shown in Figure 7A. The mean trajectories were more straightly directed toward the target and we had a smaller *wtv* when the state was taken into account for decoding than when it was not. For this particular session improvement is more evident when the point mass started from positions labeled as 2, 3, and 4 and when the decoder took into account also the temporal structure of the state (SD-TD decoder, green in the figure). We repeated the experiment for all the experimental sessions and for different values of the target radius, we measured the average *wtv* and we show the results in Figure 7B. When we used our state-dependent decoder, considering the temporal structure of the pre-stimulus ongoing activity, the *wtv* decreased on average of 26.24% with respect to the state-independent case. When we considered the state as time averaging pre-stimulus activity the *wtv* decreased on average of 14.33% with respect to the state-independent case.

## Discussion

A limit for the development of BMIs is the trial-to-trial variability in neural activity, which ultimately poses an upper limit to current BMI performance. This problem cannot be easily solved by increasing the number of electrodes used to record neural activity, because part of this variability is shared across neurons, and thus cannot be just averaged away across electrodes. One approach to solve such problem is to identify the reasons of this variability and try to predict it on each trial, so that its negative effects can be discounted by a decoding algorithm benefitting from this prediction. Here we evaluated the effectiveness of this procedure. We assumed that trial-to-trial variability arises from the dependence of single trial responses to stimuli on the ongoing variations of network state, we constructed decoding algorithms trained to learn this dependence, and then we computed how much does a BMI benefit from this state-dependent decoding.

We tested this state-dependent decoding algorithm in a BMI developed in our laboratory (Vato et al., 2012). We found both a general increase of decoding performance of more than 20%, which translated into all aspects of BMI performance, such as the convergence rate, variance and speed of the converging trajectories. The BMI equipped with a state-dependent decoder generated shorter, more accurate and stable trajectories of the simulated point mass moving toward the target with respect to the state-independent version. These advantages were obtained without the need to increase the invasiveness of the BMI (for example by increasing the number of electrodes), and came only at the moderate computational cost of including the time course of ongoing activity prior to the stimulus into our decoding algorithms.

We found that estimating network state by examining the time course of neural activity prior to stimulation, rather than relying only on time-averaged pre-stimulus activity, doubled the information gained by using state-dependence for decoding. These results suggest that the detailed time course of ongoing activity carries important information about changes in excitability of the network and strongly influences the responses to stimuli, as also put forward by recent studies using dynamical systems to model ongoing activity (Curto et al., 2009; Safaai et al., 2015).

State dependence of neural responses reflect a variety of phenomena, from the dependence of neural responses on the ongoing activity of the same network, to dependence of neural responses on changes of behavioral states such as attention or arousal that are mediated by neuromodulation. A recent study (Safaai et al., 2015) examined the interplay between ongoing activity and neuromodulation in modulating neural responses to stimuli, and found that these channels acted complementarily to determine the cortical responses to sensory stimuli. In particular, prediction of single-trial neural responses to stimuli and the decoding of the sensory information that these responses carry was much better when using knowledge of the activity of neuromodulatory nuclei as well as knowledge of ongoing activity. An implication of this finding is that the performance of state dependent decoding algorithms is expected to increase even further if including an estimate of the activity of neuromodulatory nuclei into the state dependent decoder. Interesting, the activity of such nuclei, particularly of the ones releasing norepinephrine, correlates well with pupil size with good time resolution (Costa and Rudebeck, 2016; Joshi et al., 2016), suggesting non-invasive ways to improve state-dependent BMI.

For technical reasons, we limited the exploration of the advantages of state dependence only for the decoding part of BMIs, and we could not test their potential advantages for the encoding part. One could in principle design a state-dependent encoding algorithm that, taking into account the ongoing pre-stimulus activity, modulates the stimulation parameters according to the predicted stimulus-evoked responses. Support for this suggestion was given by Brugger and colleagues that demonstrated how to use low-frequency pre-stimulus activity to increase the reliability of the neural responses in rat's somatosensory cortex (Brugger et al., 2011). Thus, closed loop BMIs that take advantage of state dependence both at the encoding and decoding stage could lead to a much larger advance in performance than the one reported here.

For experimental convenience, in this article we performed our initial attempt to improve BMI performance by exploiting state dependence only in anesthetized subjects. The obtained results suggest a benefit for BMI function when including state dependence. However, to prove that these advantages can be translated to BMI of actual use in real-life situations, it will be necessary to explore in future studies how to take advantage of state dependence in chronic BMIs implanted in awake behaving animals. Studies of state dependence of neural responses across different behavioral states suggest that this may be possible. In fact, studies of shared trial-to-trial variability across neurons have shown that such shared variability can be described by a small set of state-parameters across a variety of behavioral states, including awake behaving behavioral states (Ecker et al., 2014; Goris et al., 2014; Kayser et al., 2015; Rabinowitz et al., 2015), suggesting that this shared variability may be discounted also in awake preparation after characterizing the state dependence of their neuronal responses. However, although phase or power low frequency (1–10 Hz) fluctuations of pre-stimulus neural activity seem to have a comparable effect on post-stimulus responses both in anesthetized and awake animals (Lakatos et al., 2005; Kayser et al., 2015), the nature of state dependence may vary across different behavioral states (Ecker et al., 2014; Rabinowitz et al., 2015) meaning that both the variables describing past neural activity and the specific algorithms used to predict state dependence of post-stimulus responses need to be calibrated in each specific BMI.

A major obstacle to be overcome to successfully implement in awake animals and real-life situations state-dependent BMIs of the type conceptualized here, is that this approach in its present form is based on observing pre-stimulus activity for relatively long periods of several hundred milliseconds. This may greatly limit the frequency by which the user may interrogate and use the interface. However, the same principles invoked here can be directly extended to predict the variability of single-trial responses to frequent, dynamic stimulations, from the combined observation of the fluctuations of past neural excitability both in response to recent stimuli and in relatively inter-stimulus periods. We thus speculate that real-life situations with frequent stimulation and interrogation of neural circuits by the BMI, may benefit from the principles of state dependency by building algorithms that estimate online the current state or current excitability level of the neural circuit both using responses to recent stimuli and ongoing activity observed in the recent past in inter-stimulus intervals. These estimates of current excitability, though not directly carrying information about the external variables that the BMI seeks to sense or control, may be useful as internal state variables to interpret the neural responses to the variables to be controlled.

Addressing these issues poses technological as well as mathematical challenges. One challenge regards how to implement such algorithms in a fast online form. In this respect, we note that the mathematical functions we used here are suitable to be implemented in programmable hardware and can be even approximated by using artificial neural networks (Hinton and Salakhutdinov, 2006; Matsumoto et al., 2016), suggesting that they could be in principle implementable in adaptive and low-power consumption devices such as neuromorphic circuits (Chicca et al., 2014; Qiao et al., 2015; Boi et al., 2016). Another technological challenge regards how to track down online the temporal dynamics of pre-stimulus activity, which we showed to be useful to increase state-dependent information. In our study we tracked the time course of pre-stimulus activity by customized non-uniform temporal binning. However, this may not be practical in real life situations. Recent progresses in designing adaptive neuromorphic architectures suggest (Wang et al., 2013) that in the next future will be possible to overcome this problem by implementing adaptive temporal binning schemes in hardware. By using such adaptive architectures, it may be possible to design acquisition hardware that self-adjusts its inherent parameters (for instance, the working frequency of the sample-and-hold circuit) naturally following the neural activity variability. A next step of this research could thus be to implement our state-dependent decoding algorithm by using the neuromorphic hardware decoder developed by our group (Boi et al., 2016) and to test it with behaving rats in the experimental paradigm described in Boi et al. (2015b). A second challenge, when implementing state-dependent principles in closed-loop bidirectional BMIs (that both encode information in the nervous system by perturbing neural activity and that decode information from recorded neural activity in a closed loop) is that of the potentially different time scales of state variables (that may require observing past activity over periods of hundreds of milliseconds) and of the task- or stimulus-informative neural response variables (that often vary in short time scales of few tens of ms; Panzeri et al., 2010). This potential mismatch in time scales poses important technological challenges for implementing a closed-loop state-dependent BMI. In particular, given that electrical microstimulation produces artifacts that may mask the recorded neural signals for few ms, it is likely that running in real-life a state-dependent closed-loop BMIs will need the development of clever strategies for optimizing the time multiplexing strategy for the simultaneous stimulation readout of signals at multiple time scales (O'Doherty et al., 2011).

## Author contributions

All authors listed have made substantial, direct and intellectual contribution to the work, and approved it for publication.

### Conflict of interest statement

The authors declare that the research was conducted in the absence of any commercial or financial relationships that could be construed as a potential conflict of interest.
