# Supplementary material for: State-Dependent Decoding Algorithms Improve the Performance of a Bidirectional BMI in Anesthetized Rats
Source: Front Neurosci. 2017 May 31;11:269. doi: 10.3389/fnins.2017.00269 (PMC5449465; doi:10.3389/fnins.2017.00269)
Supplement: Supplementary file 1 [file Presentation1.PDF]

# *Supplementary Material*

# State-dependent decoding algorithms to improve the performance of a bidirectional BMI in anesthetized rats

Vito De Feo*, Fabio Boi, Houman Safaai, Arno Onken, Stefano Panzeri† and Alessandro Vato†

*** Correspondence:** Corresponding Author: vito.defeo@iit.it† *These two authors gave equal senior author contribution*

## S1 Text. State-dependent bidirectional BMI

In this paper we developed a state-dependent bidirectional BMI obtained by including a state-dependent decoder in the Dynamic Neural Interface described in (Vato et al., 2012). As in the previous work, the dynamical system was represented by a point mass moving over a horizontal plane within a viscous medium. The movement of the simulated object, obtained by applying the decoded force vector, was computed by integrating the equation of motion of the point mass in a viscous medium, $\mathrm{M}\ddot{\mathrm{x}} + \mathrm{B}\dot{\mathrm{x}} = \mathrm{F}$ , where $x = [x_1, x_2]^T$ indicates the position of the point mass on a plane. The values of mass *M* and viscosity *B* were set to 10 Kg and 15 N•s/m, respectively. We simulated this dynamics equation for a period of 1 s using standard numerical integration algorithms.

## S2 Text. Discretization and time binning of the state variables

As reported in Materials and Methods, in Sec. 2.3.1, we used different time bin sizes according to the distance time from the stimulus using 39 time bins organized as shown in **Table S1.**

To check that the pre-stimulus time windows used throughout the experiments and reported in Table S1 were optimal, we extended the pre-stimulus window to the maximum possible value, i.e. from the end of the evoked response of the previous trial to the stimulus onset. However, in this control analysis the decoding algorithm never selected neural activity in a time window starting earlier than 1.025 seconds before the stimulus onset (See Figure S1 and Table S1). This suggests that all relevant state information was contained within the [-1.025, 0] s pre-stimulus window that we used throughout the paper.

For each time bin *t*, we discretized the value of the state variable $\boldsymbol{\theta}^m(t)$ in $l \in \{1,2,3,4\}$ levels in order to have a smaller number of possible states. We optimized the value of *l* (see S4 Text) to

maximize decoding performance. Indeed, if $l$ was too small we lost information about the response trial-to-trial variability carried by the state, and the decoding performance decreased. If $l$ was too large we introduced noise into the decoder, and its performance decreased.

The cartoon on the left of **Figure 2** shows $\mathbf{\Theta}^{\mathrm{SUA},m}$ and $\boldsymbol{\theta}^{\mathrm{TA},m}$ (defined below) for a generic trial $m$. For simplicity, in the cartoon we considered $\mathbf{\Theta}^{\mathrm{SUA},m}$ as a continuous function of time, without considering the time binning and neglecting the discretization in $l$ levels.

**S3 Text. Using ongoing MUA and pre-stimulus spike as state**

MUA was obtained by pooling together the spike trains of all recorded units. For each trial $m$ and for each time bin $t$ , we obtained $\theta^{\mathrm{MUA},m}(t) \in \mathbb{R}$ by summing $\boldsymbol{\theta}^{\mathrm{SUA},m}(t) \in \mathbb{R}^{N\mathrm{x}1}$ across the rows.

We defined MUA state variables vector as,

$$\boldsymbol{\theta}^{\mathbf{MUA},\boldsymbol{m}} = [\boldsymbol{\theta}^{\mathbf{MUA},\boldsymbol{m}}(-\boldsymbol{T_\theta}), \dots, \boldsymbol{\theta}^{\mathbf{MUA},\boldsymbol{m}}(-\boldsymbol{t}), \dots, \boldsymbol{\theta}^{\mathbf{MUA},\boldsymbol{m}}(-\mathbf{1})] \in \mathbb{R}^{\boldsymbol{T_\theta}} \qquad \textbf{(S 1)}$$

We built the MUA state variables matrix, replicating $\boldsymbol{\theta}^{MUA,m}$ $N$ times and shaping any replication as a row of the matrix

$$\mathbf{\Theta}^{\mathbf{MUA},\boldsymbol{m}} = \begin{bmatrix} \boldsymbol{\theta}^{\mathbf{MUA},\boldsymbol{m}}(-\boldsymbol{T_\theta}) & \cdots & \boldsymbol{\theta}^{\mathbf{MUA},\boldsymbol{m}}(-\mathbf{1}) \\ \vdots & \ddots & \vdots \\ \boldsymbol{\theta}^{\mathbf{MUA},\boldsymbol{m}}(-\boldsymbol{T_\theta}) & \cdots & \boldsymbol{\theta}^{\mathbf{MUA},\boldsymbol{m}}(-\mathbf{1}) \end{bmatrix} \in \mathbb{R}^{\boldsymbol{N}\mathbf{x}\boldsymbol{T_\theta}} \qquad \textbf{(S 2)}$$

All the rows of this matrix were identical and, thus redundant. In this way $\mathbf{\Theta}^{\mathrm{MUA},m}$ had $N$ rows, like $\mathbf{\Theta}^{\mathrm{SUA},m}$, and this led to the advantage explained below.

For each trial $m$ we defined as MUA state activity matrix,

$$\mathbf{A}^{\mathbf{MUA},\boldsymbol{m}} = \{\mathbf{\Theta}^{\mathbf{MUA},\boldsymbol{m}}|\mathbf{R}^{\boldsymbol{m}}\} = \left\{ \begin{bmatrix} \boldsymbol{\theta}^{\mathbf{MUA},\boldsymbol{m}}(-\boldsymbol{T_\theta}) & \cdots & \boldsymbol{\theta}^{\mathbf{MUA},\boldsymbol{m}}(-\mathbf{1}) \\ \vdots & \ddots & \vdots \\ \boldsymbol{\theta}^{\mathbf{MUA},\boldsymbol{m}}(-\boldsymbol{T_\theta}) & \cdots & \boldsymbol{\theta}^{\mathbf{MUA},\boldsymbol{m}}(-\mathbf{1}) \end{bmatrix} \middle| [\boldsymbol{r}^{\boldsymbol{m}}(\mathbf{1}), \dots, \boldsymbol{r}^{\boldsymbol{m}}(\boldsymbol{t}), \dots, \boldsymbol{r}^{\boldsymbol{m}}(\boldsymbol{T_r})] \right\} \in \mathbb{R}^{\boldsymbol{N}\mathbf{x}(\boldsymbol{T_\theta}+\boldsymbol{T_r})} \qquad \textbf{(S 3)}$$

concatenating MUA state variable matrix and response matrix. This concatenation was possible because $\mathbf{\Theta}^{\mathrm{MUA},m}$ had the same number of rows as $\mathbf{R}^m$. We could apply the dimensionality reduction on the state variables matrix or vector (left part of the activity matrix) and on the response matrix (right part of the activity matrix), separately. However, we decided to apply PCA on the joint state-response matrix (activity matrix) in order to obtain a compact information rich description of both the state and the evoked activity.

We also described the network states by means of the Time Averaged pre-stimulus activity (shortened to TA). For each trial $m$, we defined the TA state variables vector as:

$$\boldsymbol{\theta}^{\mathrm{TA},m} = \frac{1}{\Delta T_{pre\_stim}} \sum_{t \in T_\theta} \boldsymbol{\theta}^m(t) \in \mathbb{R}^N \quad \text{(S 4)}$$

We defined the TA state activity matrix as

$$\mathbf{A}^{\mathrm{TA},m} = \{\boldsymbol{\theta}^{\mathrm{TA},m} | \mathbf{R}^m\} = \{\boldsymbol{\theta}^{\mathrm{TA},m} | [\boldsymbol{r}^m(1), \dots, \boldsymbol{r}^m(t), \dots, \boldsymbol{r}^m(T_r)]\} \in \mathbb{R}^{\mathrm{Nx}(1+T_r)} \quad \text{(S 5)}$$

We denoted as $\mathbf{A}^{\vartheta,m}$ the generic state activity matrix:

$$\mathbf{A}^{\vartheta,m} = \begin{cases} \mathbf{A}^{\mathrm{SUA},m} & \text{if } \vartheta = \mathrm{SUA} \\ \mathbf{A}^{\mathrm{MUA},m} & \text{if } \vartheta = \mathrm{MUA} \\ \mathbf{A}^{\mathrm{TA},m} & \text{if } \vartheta = \mathrm{TA} \end{cases} \quad \text{(S 6)}$$

We denoted as $\vartheta = SI$ the state-independent case

$$\mathbf{A}^{\vartheta,m} = \mathbf{R}^m \text{ if } \vartheta = SI \quad \text{(S 7)}$$

## S4 Text. SD-TD decoding algorithm

The decoder used the information in the confusion matrix $I(S;D)$ to optimize the following parameters:

- Neural response time window duration $\Delta T_{\mathrm{post\_stim}} \in \{100, 200, 255, 300, 400, 500, 600\}$ ms (measured as number of pre-stimulus time bins $T_r \in \{20, 40, 51, 60, 80, 100, 120\}$)
- Pre-stimulus time window duration $\Delta T_{\mathrm{pre\_stim}} \in [-1050, -5]$ ms (measured as number of pre-stimulus time bins $T_\theta \in [-39, -1]$ )
- State Variable $\vartheta \in \{\mathrm{MUA}, \mathrm{SUA}, \mathrm{TA}\}$
- Number of discretization level *l* of the state variable, $l \in \{1,2,3,4\}$
- Number of principal components $k \in [1,100]$
- Threshold value for the WTA strategy $P_{thr} \in [0.1, 0.5]$

Note that in the SD-TD decoding algorithm $\vartheta$ could be one of $\mathrm{MUA}, \mathrm{SUA}, \mathrm{TA}$. The algorithm could also choose the TA as state variable in order to include all aspects of the time dependence of pre-stimulus activity, including both the vector with the activity at any given time and the time averaged pre-stimulus activity.

Here we describe step-by-step the state-dependent decoding algorithm that we used:

1. We considered the state-independent case ( $\vartheta = SI$, $\mathbf{A}^{\vartheta,m} = \mathbf{R}^m$) and we calculated the information in the confusion matrix $I(S;D)$, without considering any state information, for all $T_r \in \{20, 40, 51, 60, 80, 100, 120\}$ . We selected the value of $T_r$ that maximized $I(S;D)$ and we used this value for all of the following steps.
2. We considered the state-dependent case and we built the state-dependent activity matrix $\mathbb{A}^\vartheta$, as we described in Sec. 2.3.2.

3. We built the test set by taking one out of *M* trials, following a leave-one-out cross-validation procedure.
4. On the training set, composed of *M*-1 remaining trials we used a further leave-one-out cross-validation calculating the values of the parameters $(P_{thr}, k, \vartheta, l, T_\theta)$ that maximized the information $I(S; D)_{training\_set}$ in the confusion matrix $\mathbf{Q}_{training\_set} \in [0,1]^{4x4}$ computed on training set (see details on this point below).
5. We used the parameters found in step 4 to decode the test trial *m* that we set aside in point 3.
6. We repeated the procedure from point 3 selecting the second trial and so on.
7. For all trials *m* we built the confusion matrix $\mathbf{Q} \in [0,1]^{4x4}$ and the information $I(S; D)$ in the confusion matrix.

We computed both the confusion matrices $\mathbf{Q}_{training\_set}$ and **Q** from the posterior probabilities, eventually applying a WTA strategy, as explained in Sec. 2.3.2 and 2.3.3.

Here we explain more extensively point 4 of the parameters' optimization procedure:

a. We set $\vartheta = MUA$ and $l = 2$ .
b. For each of the following values of $T_\theta = 1, \ldots, 39$ we calculated the couple $(P_{thr}, k)$ that maximized $I(S; D)_{training\_set}$. We varied $k$ in the range $[1{,}100]$ and $P_{thr}$ in the range $[0.1{,}0.5]$. We recorded the best combination $(T_\theta, P_{thr}, k)$.
c. We repeated step b and c for each of the following couples of parameters:
   d1. $\vartheta = \text{MUA and } l = 2$
   d2. $\vartheta = \text{MUA and } l = 3$
   d3. $\vartheta = \text{MUA and } l = 4$
   d4. $\vartheta = \text{SUA and } l = 2$
   d5. $\vartheta = \text{TA and } l = 4$
   d6. $\vartheta = \text{SI}$
d. We chose the values of $P_{thr}, k, \vartheta, l$ and $T_\theta$ which maximize $I(S; D)_{training\_set}$.

   For $\vartheta = SUA$, we used two discretization levels ($l = 2$) because we did not gain information by using more levels. Indeed by using $l > 2$ we introduced noise, decreasing decoding performance. We applied an analogous procedure for the MUA and for the TA. We did analogous considerations also for the parameters $k, T_\theta$ and $T_r$. If their values were too small, we lose information, if they were too large we introduced noise. Our algorithm found the best compromise. As shown in **Figure S1A** we had $\vartheta$ = SUA, $\vartheta$ = MUA, $\vartheta = TA$ in 47%, 10.5% and 42.5% of the trials, respectively. **Figure S1B** shows the cumulative distribution of $T_\theta$, measured in number of time bins (See Table 1), averaged across all trials and all experimental sessions.

Using the temporal structure of the pre-stimulus ongoing activity we approximatively doubled the decoding performance with respect to the SC case. On the other hand a state-dependent algorithm

that considers only the spike count of the pre-stimulus activity is simpler and faster (2-3 times faster) because it skips steps d1, d2, d3 and d4.

## *Supplementay Figures and Tables*

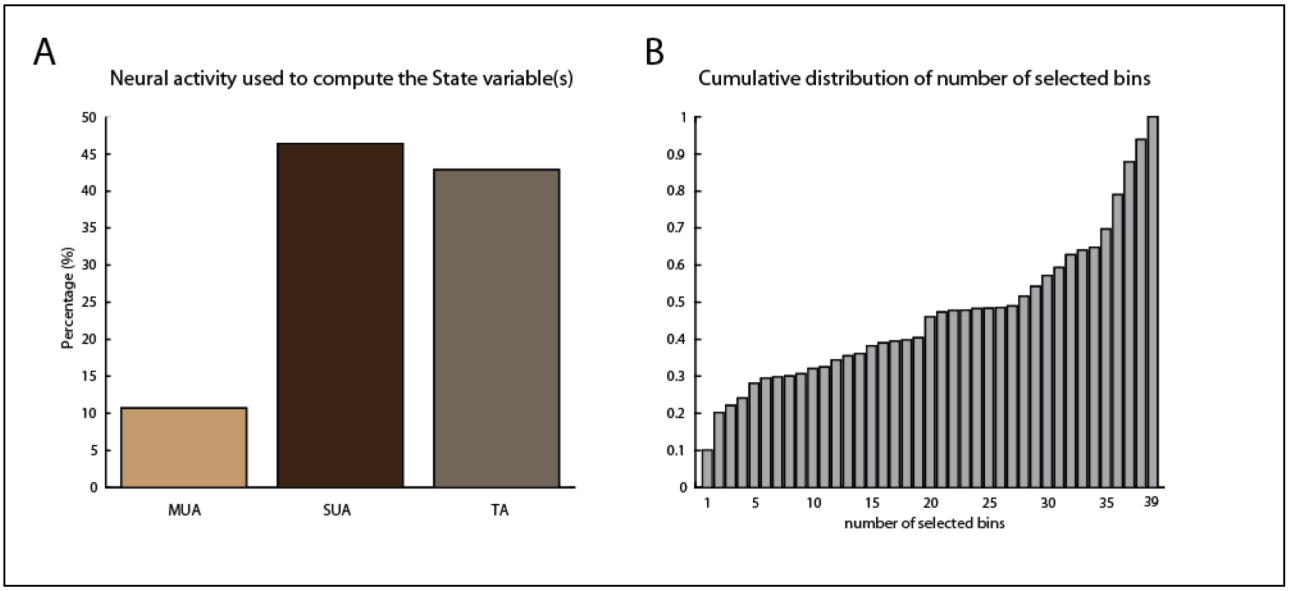


**Figure S1**: (A) The graph shows the percentage of instances when the algorithm selected the different signals (MUA, SUA and TA) to compute the state variable(s) across all experimental sessions (B) Cumulative distribution of the number of the pre-stimulus time bins selected for inclusion by the algorithm across the whole dataset.

| bin label(s) | bin duration | time interval (ms) |
|---|---|---|
| [-1 ÷ -30] | 5 ms | [-150 ms, 0 ms] |
| -31 | 25 ms | [-175 ms , -150 ms] |
| -32 | 50 ms | [-225 ms, -175 ms] |
| [-33 ÷ -35] | 100 ms | [-525 ms, -225 ms] |
| [-35 ÷ -39] | 200 ms | [-1025 ms, -225 ms] |

**Table S1**. Pre-stimulus time bins labels, durations and correspondent pre-stimulus time intervals (0 corresponds to the stimulus onset).
